# Management of Transplant Renal Artery Pseudoaneurysm and Literature Review

**DOI:** 10.1155/2022/6232586

**Published:** 2022-06-11

**Authors:** Luke Anders, Rachel Stephens, Melissa Laub, Rushay Amarath-Madav, Ahmad Mirza, Muhammad Irfan Saeed

**Affiliations:** ^1^Medical College of Georgia at Augusta University, Augusta, GA, USA; ^2^Department of Pharmacology, Medical College of Georgia at Augusta University, Augusta, GA, USA; ^3^Department of Surgery, Medical College of Georgia at Augusta University, Augusta, GA, USA

## Abstract

Renal transplantation is the ultimate treatment for end-stage renal disease patients. However, vascular complications can impact renal allograft outcomes. Extrarenal pseudoaneurysms (EPSA) are a rare complication occurring in 1% of transplant recipients. We report a case series of extrarenal pseudoaneurysm after kidney transplant with different clinical presentations and management strategies. Given the rarity of EPSA, literature describing this complication is limited to single case reports or small retrospective case series. We also provide an up-to-date review of 76 articles on mycotic, bacterial, and idiopathic EPSAs. Allograft removal is considered standard treatment, but new endovascular alternatives may allow allograft salvage. EPSA should be managed with a multidisciplinary approach. Surveillance with renal ultrasound is recommended in patients considered high risk.

## 1. Introduction

Vascular complications, which have an incidence rate of 6-30% after a kidney transplant, can impact allograft and patient outcomes [1]. Vascular complications include renal artery stenosis, arterial/venous thrombosis, arteriovenous fistulas, and renal artery pseudoaneurysms [1]. Intrarenal pseudoaneurysms are most commonly secondary to percutaneous kidney biopsy, infection, or technical error but usually resolve spontaneously with time, despite a 5% occurrence rate [2]. Extrarenal pseudoaneurysms (EPSAs) carry an incidence rate of 1% and cause irreversible arterial destruction of the vessel wall; causes can include infectious and noninfectious etiologies [1, 3, 4]. Despite their rarity, EPSAs are a serious complication, which profoundly impact both graft and patient survival. Though small EPSAs can be closely observed, large or infected EPSAs are a clinical emergency. Rupture of the EPSA creates life-threatening bleeding, so allograft nephrectomy, either prophylactically or emergently, is the recommended treatment of choice [5–7]. Evidence-based care of this complication is limited to single case reports or small retrospective series. Here, we report three cases of infectious EPSA after kidney transplantation, including their wide-ranging clinical presentations, treatments, and outcomes (Table 1). Additionally, we present an up-to-date review of the literature to provide the reader with a multitude of examples of how this anomaly may present in practice (Table 2).

## 2. Case Presentation

### 2.1. Case 1 (Figure 1)

The patient was a 68-year-old male with end stage renal disease (ESRD) secondary to Wegener's granulomatosis who received his second deceased donor kidney transplant (DDKT) in November 2019. His postoperative course was significant for delayed graft function secondary to acute tubular necrosis (ATN). On postoperative day (POD) 19, he presented to clinic with complaints of fatigue, chills, and rigors. He was diagnosed with urosepsis. Both blood and urine cultures on admission grew *Pseudomonas aeruginosa*. Patient was started on broad spectrum antibiotics which included clindamycin, piperacillin/tazobactam, then cefepime and tobramycin at various points.

A noncontrast computer tomography (CT) scan of the abdomen pelvis on admission was negative for any peri-nephric fluid collection. Overnight, he acutely decompensated and coded. Advanced cardiac life support protocol was initiated with return of spontaneous circulation after 20 minutes. Repeat CT angiogram (CTA) of the abdomen/pelvis was notable for a large retroperitoneal hematoma with active bleed from EPSA of the donor renal artery. Interventional radiology (IR) was consulted for placement of a covered stent. This intervention was unsuccessful, and ultimately, the patient was taken to the operating room and underwent transplant nephrectomy and saphenous vein patch angioplasty of the external iliac artery. The donor renal artery was completely avulsed from the recipient artery at the anastomosis site.

The patient remained in the intensive care unit (ICU) postoperatively secondary to septic shock and multiorgan failure. On POD 39, he died of septic shock secondary to ischemic bowel and bowel perforation. Interestingly, the liver recipient from the same cadaveric donor, who was transplanted at another institution, also died suddenly at home.

### 2.2. Case 2 (Figure 2)

The patient is a 63-year-old male with ESRD secondary to diabetes and hypertension who received a DDKT in January 2020. The patient's immediate postop course was complicated by wound infection and dehiscence requiring multiple wash outs and biological mesh repair. Almost six months posttransplant, he was presented to a routine clinic follow-up with 1 week history of right flank pain, elevated blood pressure, hematuria, and a leaking sinus at his Gibson incision. Labs were significant for anemia and acute kidney injury with serum creatinine of 7 mg/dL from a baseline of 2 mg/dL.

Ultrasound showed a juxta-anastomotic pseudoaneurysm measuring 5 × 5 cm confirmed on CT with concern for a contained anastomotic leak. The patient was admitted to ICU and started on broad spectrum antimicrobials empirically, which included metronidazole, vancomycin, and micafungin. Blood and urine cultures from admission grew *Enterococcus faecalis*. Vascular surgery performed an angiogram that showed an anastomotic pseudoaneurysm, but it was deemed unsafe to coil due to a wide neck.

After detailed discussion with the patient, we decided to proceed with a Gore excluder cover stent placement in the external iliac artery with loss of the transplanted kidney because of the expanding pseudoaneurysm. Surgical repair option was discussed but considered too high risk for this patient and still carried a significant chance of graft loss. After stent deployment, CTA confirmed an excluded pseudoaneurysm with no evidence of leak. Transthoracic and transesophageal echocardiograms showed no vegetation or evidence of endocarditis. Repeat blood and urine cultures on hospital days 3 and 5 were negative, and his antibiotic regimen was changed to ampicillin monotherapy. The patient was restarted on hemodialysis and discharged to an inpatient rehab facility to finish a six-week antibiotic course. The patient is still alive and awaits a second kidney transplant.

### 2.3. Case 3 (Figure 3)

The patient is a 69-year-old female who received a DDKT in May 2021 and presented to clinic in July 2021 complaining of dysuria and pain at her Gibson incision. Her urine culture showed >100,000 CFU/mL vancomycin-resistant *Enterococcus faecium* (VRE). Labs were significant for a drop in hemoglobin to 8.7 g/dL from 11.2 g/dL. She had experienced four prior urinary tract infections (UTIs) since transplant with unclear etiology; previously urine cultures demonstrated *Candida tropicalis*, VRE, and *Pseudomonas aeruginosa* organisms, which were appropriately treated with active antimicrobials. Notably, she never had positive blood cultures at any point in her course. At this clinic visit, she was readmitted for treatment of the current VRE infection with daptomycin per infectious disease recommendations, given history of recurrent UTIs.

CT abdomen/pelvis on admission demonstrated a subcutaneous hematoma measuring 4 × 4 × 15 cm along her Gibson incision. Ultrasound of the transplanted kidney showed elevated peak velocities concerning for renal artery stenosis but gave no mention of pseudoaneurysm. A repeat transplant kidney ultrasound the next day showed a new onset 3 × 3 cm EPSA arising from the proximal transplant renal artery.

Angiogram demonstrated a large and small EPSA arising from the proximal and midtransplant renal artery, respectively. There was no anastomotic EPSA. The distal transplant renal artery was severely stenosed but patent. Due to nonavailability of customized cover stent, the procedure was performed in two stages. Initially, the IR team coil embolized the larger EPSA, and she returned to IR the following day for placement of two 6 mm × 20 mm covered stents with angioplasty of the distal transplant renal artery. Repeat inpatient blood cultures were negative. She was discharged home on a four-week course of linezolid and fluconazole to cover the VRE and previous *Candida tropicalis*. The patient is doing well, and her repeat urine and blood cultures have remained negative of antimicrobials.

## 3. Discussion

EPSA is a rare (1% incidence rate) but devastating complication of kidney transplantation. EPSA can occur at or adjacent to the surgical anastomosis, usually secondary to a mycotic or bacterial infection. Previous literature reviews place the incidence of allograft loss at 56%, concurrence with an infective pathogen at 62%, and mortality at 14% [8]. In multiple retrospective series, *C. albicans* was the leading pathogen in cases with infection [8–10]. The mechanism by which mycotic and bacterial pseudoaneurysms develop is well-described in the literature, involving an inflammatory process that invades and compromises the wall of the artery [11].

Patients with kidney transplants often have multiple risk factors for opportunistic infections, including immunosuppression, end-stage renal failure, diabetes mellitus, hypertension, and dyslipidemia [12–14]. Additionally, prolonged ICU stays (>7 days) and extended operative times are associated with increased incidence [15]. Intuitively, age should be a risk factor for all operative complications, but this association is not reflected in the data [16]. Procurement of organs, transportation, back-table preparation, and transplantation involve countless opportunities for inadvertent contamination; the likelihood of transplant contamination is estimated at 40% [12]. This prevalence highlights the importance of obtaining donor cultures from multiple sites, careful handling of the allograft, and strict sterile technique at all times. Organ procurement organizations (OPO) work extensively with the donor centers to ensure minimal infection risk, frequently involving infectious disease colleagues to confirm thorough treatment and prophylaxis. While we extensively scan donors, preservation fluid, and recipient blood cultures, some smoldering infections can be masked or subdued by extended courses of antibiotics, making detection of some pathogens difficult [14].

**Figure 2:**
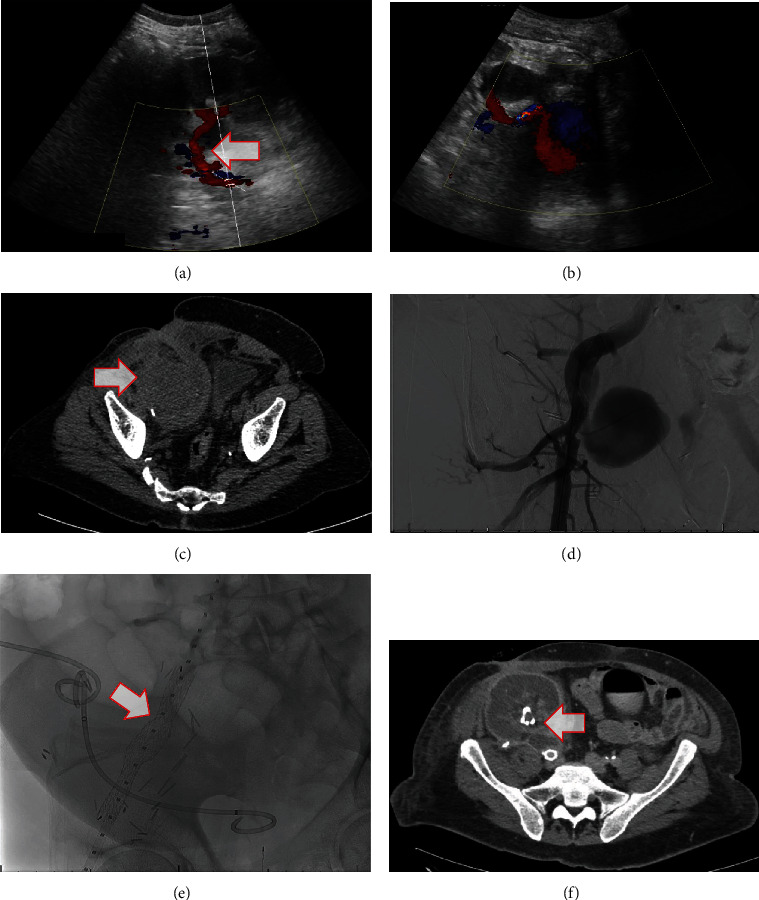
Case 2 imaging, whose blood and urine cultures on admission grew *Enterococcus faecalis*. (a) Ultrasound from initial transplant hospitalization demonstrating normal RLQ transplant kidney with patent renal artery and vein without pseudoaneurysm. (b) Ultrasound on admission demonstrating pseudoaneurysm at the anastomosis measuring 5 cm × 4 cm × 6 cm. (c) Noncontrast CT demonstrating 8 cm × 8 cm hematoma in the same area of the pseudoaneurysm seen on ultrasound approximately 4 hours earlier. (d) Arteriogram demonstrating 5 cm × 5 cm juxta-anastomotic pseudoaneurysm; it was deemed unsafe to coil given its wide neck. (e) Arteriogram on the following day after placement of a 16 mm × 14.5 mm × 10 cm Gore excluder endograft. (f) CT angiogram demonstrating endograft placement completely excluding the anastomotic pseudoaneurysm.

**Figure 3:**
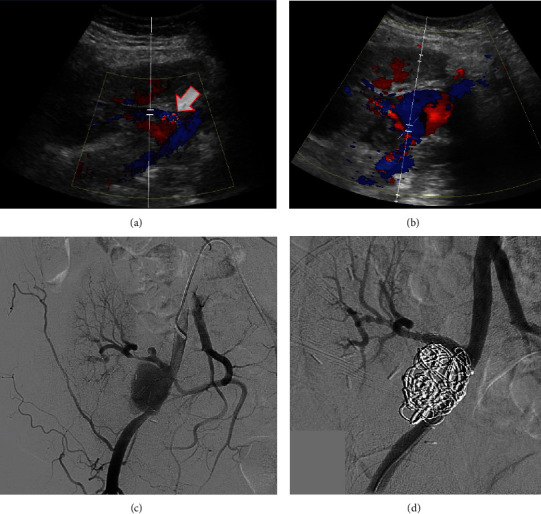
Case 3 imaging, whose urine cultures on admission grew vancomycin-resistant *Enterococcus faecium*. She had 4 UTIs since her transplant with unclear etiology; previous UTIs included VRE, *Pseudomonas aeruginosa*, and *Candida tropicalis*. (a) Ultrasound 1 month prior to admission demonstrating normal RLQ transplant kidney with patent renal artery and vein without pseudoaneurysm. (b) Ultrasound on admission demonstrating 3 cm × 3 cm pseudoaneurysm arising from the renal transplant artery. (c) Arteriogram on the following day demonstrating a large and small pseudoaneurysm arising from the proximal and midtransplant artery, respectively. Severe stenosis also seen at the distal transplant artery. (d) Arteriogram demonstrating exclusion of both pseudoaneurysms as well as angioplasty of the distal transplant artery.

The clinical presentation of EPSAs varies considerably, evident both within our own cases and the literature. Patients can be totally asymptomatic (Case 3) or acutely decompensating from aneurysmal rupture (Case 1). Time to diagnosis varied both within our own case series as well as cases in literature, ranging from a few days to many years after the initial transplant. Other symptoms include pulsatile masses, abdominal tenderness, lower limb ischemia, allograft dysfunction, anemia, or signs of infection [4, 15, 17]. Doppler ultrasonography is the first imaging tool used, ideally showing the pathognomonic “Yin Yang” sign indicating turbulent mixing of blood as exhibited in Figures 2(b) and 3(b). CT or magnetic resonance (MR) angiography can confirm the pathology as well as evaluate its impact on surrounding structures such as the ureter or iliac vessels [18]. Conventional angiography elucidates the exact location and occasionally allows for simultaneous percutaneous treatment.

All three of our cases presented with an associated infection. In Case 1, *Pseudomonas aeruginosa* grew in urine and blood cultures; per literature review, this is the most frequent bacteria associated with EPSA occurrence [8, 11]. Rapidly developing an EPSA in less than 3 weeks, Case 1 is a good reminder that all infections, especially pathogens historically linked to the formation of EPSAs such as Pseudomonas, should trigger aggressive, immediate treatment, and surveillance after antibiotic completion. Case 2 is notable as *Enterococcus faecalis* has only been associated with EPSA in one other case in the literature. Notably, Case 3 had multiple UTIs prior to discovering the EPSA, but no positive blood cultures. In this instance, it is possible that long-term treatment for her multiple UTIs masked a developing vascular insult, at which point the EPSA was incidentally noted.

Indications for repair of an EPSA are controversial, but pseudoaneurysms with a diameter of >2.5 cm are almost uniformly at a high risk of rupture [1, 7, 15, 16, 19]. Additional intervention indications include symptom severity, rate of size enlargement, presence of infection, and renal artery hypertension [1, 15]. EPSAs smaller than 2 cm can usually be managed conservatively with serial imaging and resolve spontaneously in some cases [20]. Therapeutic options for large or high-risk EPSAs include allograft nephrectomy, conventional open repair (allograft removal, creation of new vascular anastomoses, and repair of previous site with patch angioplasty), endovascular stenting or coiling, and/or ultrasound-guided percutaneous thrombin injection [14, 21]. In both our own practice and literature review, transplant nephrectomy represented the gold standard for definitive treatment but is a tough decision. Endovascular intervention represents a promising future for repair that retains allograft function, but, as in Case 2, sometimes necessitates excluding the allograft to prevent rupture. In such scenarios, a transplant nephrectomy may be performed later based on the patient's clinical condition (development of allograft abscess, etc.). The authors, including Lin et al. and Cano-Velasco et al., have compiled past articles on this subject, and Cano-Velasco et al. proposed a treatment algorithm based on these reports [8, 16].

Management of EPSA requires a multidisciplinary approach. Patient and allograft salvage are the prime goal. Newer endovascular techniques open new options for salvaging the graft. Each case is different and needs thorough assessment and unique plan development based on the patient's needs.

## 4. Conclusion

EPSA of the transplant renal artery is a rare issue. Prevention, high degree of suspicion, and aggressive multidisciplinary management are needed to save the patient and renal allograft. Surveillance of patients with previous urinary or bloodstream infections is also recommended.

## Figures and Tables

**Figure 1:**
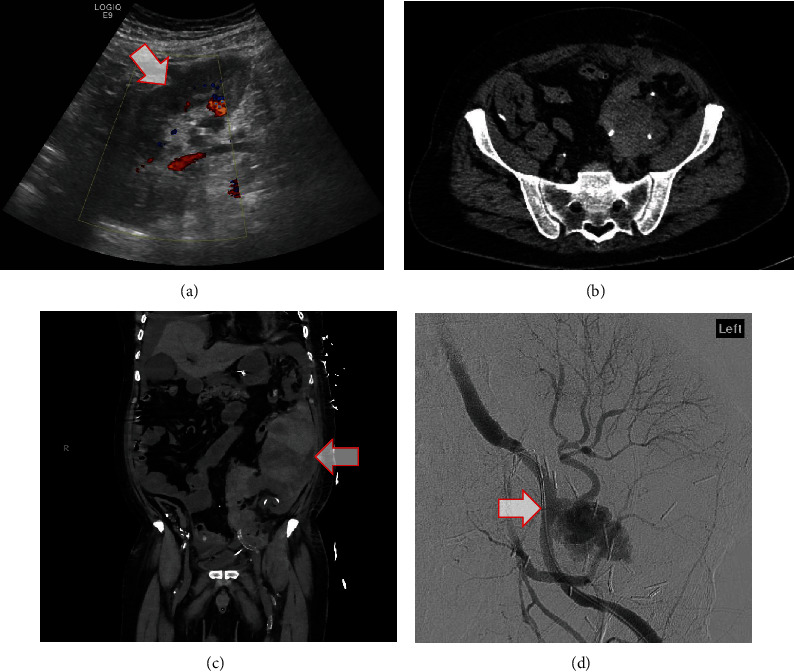
Case 1 imaging, whose blood and urine cultures on admission grew *Pseudomonas aeruginosa*. (a) Ultrasound on admission demonstrating normal LLQ transplant kidney with patent renal artery and vein without pseudoaneurysm. (b) Noncontrast CT A/P demonstrating slight interval increase in size of hematoma compared to prior imaging. No evidence of active bleeding. (c) CT angiogram demonstrating irregular, actively bleeding pseudoaneurysm arising from the transplant renal artery ~1.2 cm from the anastomosis. (d) Arteriogram demonstrating pseudoaneurysm with active contrast extravasation. A 6 mm × 22 mm covered stent was placed successfully within the proximal transplant renal artery; however, persistent hemorrhage warranted emergent surgical management.

**Table 1:** Summary of our three cases, including clinical presentations, treatments, and outcomes.

	Age	Time since transplant	Organisms cultured in urine posttransplant	Organisms cultured in blood posttransplant	Diagnosis on imaging	Outcome
Recipient 1	68	2.5 weeks	*Pseudomonas aeruginosa* (on admission)	*Pseudomonas aeruginosa* (on admission)	CTA: hematoma with active bleed from IPA of donor renal artery	Nephrectomy
Recipient 2	69	27 weeks	*Enterococcus faecalis* (on admission)	*Bacteroides* (week 4) *Enterococcus faecalis* (on admission)	Noncontrast CT: 8 × 8 cm mass highly suspicious for blood components, seen in the same area of the pseudoaneurysm seen in his initial ultrasound	Endovascular exclusion via covered stent
Recipient 3	64	6 weeks	Vancomycin-resistant *Enterococcus faecium* and *Candida tropicalis* (week 1) *Pseudomonas aeruginosa* (week 5) Vancomycin-resistant *Enterococcus faecium* (week 6)	N/A	Angiography: large and small pseudoaneurysms arising from the proximal and midtransplant renal artery, respectively	Large PA: coil embolization +6 × 16 mm covered stent Small PA: 6 × 16 mm covered stent

**Table 2:** Review of literature on extrarenal pseudoaneurysms in the PubMed/MEDLINE and Google Scholar databases (1978–September 1, 2021). Tx: transplantectomy; SR: surgical repair; EVS: endovascular stenting; EVC: endovascular coiling; OBS: observation.

Author	Year	*N*	Infection	Interval after transplant	Intervention	Outcome
Bacterial pseudoaneurysms in literature review						
Nelson	1984	1	1/*P. aeruginosa*	11 days	1/Tx	1/graft loss
Kumar	2002	1	1/*P. aeruginosa*	9 days	1/Tx	1/graft loss
Saidi	2004	2	2/*P. aeruginosa*	**—**	2/Tx	2/graft loss
Eng	2006	4	1/MRSA, C*. difficile* 1/MRSA 1/*S. marcescens* 1/*C. albicans*	**—**	4/Tx	4/graft loss 2/death
Fujikata	2006	1	1/MRSA	1.3 months	1/OBS	1/graft preserved
Nguan	2006	1	1/*S. aureus*	**—**	1/Tx	1/graft loss
Poels and Riley	2007	1	1/*P. aeruginosa*	1.7 months	1/EVS + thrombin	1/graft preserved
Orlando	2009	2	2/*P. aeruginosa*	11-21 days	2/Tx	2/death
Berglund	2011	1	1/*P. aeruginosa*	46 days	1/SR	1/graft preserved
Buimer	2012	1	1/*E. coli*	14 months	1/SR	1/graft preserved
Kaabak	2013	1	1/*P. aeruginosa*	10 days	1/SR	1/graft preserved
Chandak	2014	1	1/*P. aeruginosa*	10 days	1/SR	1/graft preserved
Che	2014	1	1/*E. coli*	4 months	1/EVS	1/graft preserved
Patil	2015	1	1/*E. coli*	21 days	1/EVS	1/graft preserved
Berger	2017	2	2/*P. aeruginosa*	3-15 days	1/Tx 1/EVS	1/graft loss 1/graft preserved
Chung	2017	1	1/*P. aeruginosa*	1 month	1/Tx	1/graft loss
Mycotic pseudoaneurysms in literature review						
Potti	1998	1	1/*C. albicans*	**—**	1/Tx	1/graft loss
Battaglia	2000	2	2/*C. albicans*	17 days-3 months	2/Tx	2/graft loss
Calvino	2003	2	2/*C. albicans*	**—**	2/Tx	2/graft loss
Garrido	2003	2	2/*A. flavus*	1.5-4 months	2/Tx	1/death 1/graft loss
Peel	2003	1	1/*C. albicans*	1 month	1/SR + EVC	1/graft preserved
Laouad	2005	4	4/*C. albicans*	9 days-3 months	4/Tx	3/graft loss 1/death
Zavos	2005	3	2/*C. albicans*	**—**	2/Tx	2/graft loss
Henderson	2007	1	1/*C. albicans*	4 months	1/Tx	1/graft loss
Liu	2009	1	1/*A. flavus*	12 months	1/Tx	1/graft loss
Osman	2009	1	1/*C. albicans*	1.2 months	1/EVS + Tx	1/graft loss
Taksin	2009	1	1/*C. albicans*	3 weeks	1/Tx	1/graft loss
Wang	2009	3	4/*A. flavus*	10 days-1.5 months	4/Tx	4/graft loss
Akhtar	2011	1	1/*C. albicans*	**—**	1/Tx	1/graft loss
Lee	2011	1	1/*C. albicans*	2 months	1/Tx	1/graft loss
Minz	2011	2	2/*A. flavus*	1-5 months	2/Tx	1/death 1/graft loss
Polat	2011	1	1/*C. albicans*	**—**	1/Tx	1/graft loss
Kountidou	2012	1	1/*C. albicans*	3 months	1/SR	1/graft preserved
Ram Reddy	2012	2	2/*A. flavus*	3-20 weeks	2/Tx	2/graft loss
Debska-Slizien	2015	2	2/*C. albicans*	10-30 days	2/Tx	2/death after OP
Madhav	2015	1	1/*C. albicans*	25 days	1/SR	1/graft preserved
Zhao	2016	2	2/*C. albicans*	14-21 days	2/EVS + Tx	2/graft loss
Lazarus	2016	1	1/*C. albicans*	47 days	1/Tx	1/graft loss
Lin	2017	2	2/*C. albicans*	14-32 days	1/Tx 1/SR	1/graft loss 1/graft preserved
Ministro	2017	2	2/*C. albicans*	60-150 days	2/SR	2/graft preserved
Mixed pseudoaneurysms in literature review						
Kyriakides	1976	8	4/*E. coli* 2/*C. albicans* 2/*P. aeruginosa*	1.5-6 months	8/Tx 1/SR	2/death 6/graft loss
Koo	1999	3	1/MRSA 2/none	2-3 months	1/EVC 1/Tx 1/OBS	1/graft loss 2/graft preserved
Bracale	2009	12	1/*E. coli* 2/*C. albicans* 9/none	13 days-49 months	8/Tx 3/EVS + Tx 1/SR + replantation	8/graft loss 3/death after OP 1/graft preserved
Bozkurt	2010	2	1/*C. albicans* 1/*E. faecalis*	11-18 days	2/Tx	2/graft loss
Leonardou	2012	4	2/*P. aeruginosa* 1/*K pneumonia* 1/*C. albicans*	3-15 months	4/EVS + Tx	4 graft loss
Santangelo	2013	6	2/*C. albicans* 4/none	1.5-10 months	1/SR + replantation 4/Tx 1/EVS + Tx	1/graft preserved 5/graft loss
Patrono	2015	3	2/*C. albicans* 1/*P. aeruginosa*	12-25 days	2/Tx 1/SR	2/graft loss 1/graft preserved
Fananapazir	2016	4	2/*P. aeruginosa* 2/none	2-12 weeks	3/Tx 1/EVC	3/graft loss 1/graft preserved
Liu	2018	5	2/*A. baumannii* 2/*C. albicans* 1/*S. epidermidis*	9-21 days	5/SR	5/graft preserved
Idiopathic pseudoaneurysms in literature review						
Renigers and Spigos	1978	1	1/none	28 days	1/Tx	1/graft loss
Benoit	1988	1	1/none	6 months	1/Tx	1/graft loss
Koo	1999	3	3/none	2-4 months	1/Tx 2/observation	1/graft loss 2/graft preserved
Reus	2002	1	1/none	2 months	1/thrombin	1/graft loss
Taghavi	2005	1	1/none	72 months	1/SR	1/graft preserved
Zavos	2005	2	2/none	5 months	2/EVS	2/graft loss
Asztalos	2006	1	1/none	6 months	1/SR	1/graft preserved
Fujita	2006	1	1/none	5 months	1/EVS	1/graft preserved
Siu	2006	1	1/none	3 months	1/EVS + thrombin	1/graft preserved
Fornaro	2007	1	1/none	15 months	1/thrombin	1/graft preserved
Gravante	2008	1	1/none	6 months	1/SR	1/graft preserved
Orlic	2008	1	1/none	2.5 months	1/Tx	1/graft loss
Sharron	2009	1	1/none	3 months	1/SR + thrombin	1/graft preserved
Al-Wahaibi	2010	1	1/none	4 months	1/SR	1/graft preserved
Akgul	2011	1	1/none	14 years	1/EVC	1/graft preserved
Favelier	2012	1	1/none	36 months	1/EVC and stent	1/graft preserved
Smeds	2013	1	1/none	72 months	1/EVS	1/graft preserved
Tshomba	2015	1	1/none	9 months	1/EVS	1/graft preserved
Ardita	2015	1	1/none	20 days	1/SR	1/graft preserved
Farooqui	2016	1	1/none	2 months	1/SR	1/graft preserved
Turunc	2017	1	1/none	1 month	1/EVS	1/graft preserved
Marie	2018	1	1/none	5 months	1/EVC	1/graft preserved
Sharma	2018	2	2/none	14-24 months	1/SR 1/EVS	2/graft preserved
Ugurlucan	2018	1	1/none	3 months	1/EVC	1/graft preserved
Haijie	2020	6	6/none	—	6/EVS	3/graft loss 3/graft preserved
Vijayvergiya	2021	1	1/none	—	1/EVC and stent	1/graft preserved
Xu	2021	1	1/none	6 months	1/observation	1/graft preserved

## Data Availability

The articles referenced in this publication are available online through a multitude of article databases and publisher websites.
